# Structural Neuroimaging and Molecular Signatures of Drug-Naive Depression With Melancholic Features

**DOI:** 10.1155/2024/9680180

**Published:** 2024-10-14

**Authors:** Lijin Yuan, Zhaosong Chu, Xianyu Chen, Mengxin He, Yi Lu, Xiufeng Xu, Zonglin Shen

**Affiliations:** ^1^Department of Psychiatry, First Affiliated Hospital of Kunming Medical University, Kunming 650032, Yunnan, China; ^2^Department of Medical Imaging, First Affiliated Hospital of Kunming Medical University, Kunming, Yunnan 650032, China; ^3^Yunnan Clinical Research Center for Mental Disorders, Kunming 650032, China

**Keywords:** major depressive disorder, melancholic features, neurotransmitter receptor, precuneus, voxel-based morphometry

## Abstract

**Objectives:** Melancholic depression (MD) is a common subtype of major depressive disorder (MDD). It is difficult to treat because its neurobiological basis is poorly understood. Therefore, to investigate whether MD patients have any structural changes in gray matter (GM) and the molecular foundation of these changes, we combined voxel-based morphometry (VBM) analysis with neurotransmitter system-derived mapping from public data.

**Methods:** 137 drug-naive MDD patients and 75 healthy controls (HCs) were recruited for structural magnetic resonance imaging. The imaging results were analyzed using VBM analysis. MDD patients were then divided into MD and nonmelancholic depression (NMD) subgroups according to their scores on the Montgomery–Asberg Depression Rating Scale (MADRS) and the Hamilton Depression Rating Scale. Next, we analyzed the spatial correlation between the changes in the gray matter volume (GMV) maps and the neurotransmitter receptor/transporter protein density maps provided by the JuSpace toolbox.

**Results:** Compared to HCs, patients with MD had significant GMV reduction in the bilateral hippocampus, bilateral thalamus, right amygdala, and right posterior cingulate cortex (PCC)/precuneus. Compared to patients with NMD, MD patients had significant GMV reduction in the bilateral PCC/precuneus and lateral occipital cortex. Moreover, compared to HCs, changes in GMV introduced by MD were spatially associated with the serotonin transporter, cannabinoid receptor, and *μ*-opioid receptor. Compared to NMD patients, changes in GMV introduced by MD were spatially associated with the vesicular acetylcholine transporter.

**Conclusion:** The present study discovered abnormal GMV alterations in patients with subtypes of depression. We also found a series of neurotransmitter receptors that may be associated with the alterations. The findings of the current study may provide a more comprehensive understanding of the molecular mechanisms underlying the structural abnormalities in subtypes of depression and potentially offer new insights into developing new therapeutic strategies.

## 1. Introduction

Melancholic depression (MD) is the most common type of major depressive disorder (MDD) [1]. It is characterized by anhedonia, psychomotor disturbance, excessive guilt or hopelessness, suicidal features, and changes in appetite or weight [2, 3]. Patients with MD usually have more severe clinical manifestations than patients with nonmelancholic depression (NMD), such as an earlier age of onset [2], more suicidal ideations [4], and more severe cognitive impairment [5]. Because the distinct clinical features of MDD are associated with different neurobiological signatures of the brain, MD and NMD may have different pathogenesis [6, 7].

The voxel-based morphometry (VBM) technique can accurately measure changes in gray matter volume (GMV), which can be used to explore the pathological mechanisms of depression. Previous studies using structural neuroimaging found MDD patients had abnormal GMV. A more recent VBM meta-analysis discovered significant GMV reductions in the bilateral cerebellum and the left inferior frontal gyrus (IFG) among MDD patients compared to healthy controls (HCs). After applying a more liberal threshold, the reduction extended to the right IFG (including bilateral insula), left superior frontal gyrus (including anterior cingulate subregions and right ventromedial prefrontal cortex), bilateral hippocampi, and left rolandic operculum (including right posterior cingulum) [8]. However, only a few studies focused on the changes in GMV in MD or NMD patients, with inconsistent findings. For instance, previous studies have reported that compared to patients with NMD, patients with MD had reduced hippocampal volume [9]. In contrast, another study did not find any significant differences in hippocampal volume between patients with MD and patients with other subtypes of MDD. However, they did find that the larger amygdala volume in MD patients compared to NMD patients [10]. Moreover, previous studies using structural neuroimaging have not revealed the molecular basis of the structural abnormalities in MDD, nor have they reach a conclusion about the underlying neurophysiological mechanisms behind the abnormalities.

The biological etiology of MDD is closely related to the deficiency of monoamine neurotransmitters (including serotonin, norepinephrine, and dopamine) and the functional abnormality of neurotransmitter receptors. Meanwhile, disruptions of the monoamine neurotransmitter system may exist in different neural circuits in different brain regions [11]. Moreover, previous studies suggested that GMV may also be influenced by neurotransmitter transporters/receptors [12]. A review article indicated that serotonin is a vital neuromodulatory factor regulating neuroplasticity during early development, and changes in serotonergic neuroplasticity may lead to reduced neuronal density and size, and reductions in hippocampal volume in MDD patients [13]. However, dysfunction of the serotonergic system alone is not sufficient to fully elucidate the pathogenesis of depression [14, 15]. The new therapeutic direction for depression modulating the GABA and glutamate systems also suggests that the pathological mechanisms of depression involve changes in multiple neurotransmitter systems [16, 17]. Therefore, we combined multiple neurotransmitter systems to identify the neurotransmitter systems associated with changes in the GMV in MD or NMD patients. The JuSpace toolbox (https://github.com/juryxy/JuSpace) is a tool for spatial correlation analysis of MRI data with nuclear imaging-derived neurotransmitter maps from public data. It can also link neuroimaging to underlying neurotransmitter information [18]. To date, it has been used to explore the spatial correspondence between neurotransmitter receptors/transporters and structural MRI abnormalities for diseases including depression [19] and schizophrenia [20, 21].

In the present study, we aim to investigate brain structural abnormalities in patients with MD. We will also combine the results with information obtained on molecular features to fill the gap between the molecular mechanisms and macroscopic neuroimaging phenotypes of drug-naïve MD patients.

## 2. Methods

### 2.1. Participants

We recruited 137 first-episode and drug-naive patients with MDD (aged 18–60) from the outpatient and inpatient departments of the First Affiliated Hospital of Kunming Medical University from February 2012 to July 2015. Two psychiatrists independently diagnosed patients using the mood disorders component of the Structured Clinical Intervie for DSM-IV Axis I Disorders. We included patients who: (1) were newly diagnosed with major depression and scored 12 or more on the Montgomery Depression Rating Scale [22] and 17 or more on the Hamilton Rating Scale for Depression (HDRS_−17_) [23]; (2) did not have a history of mental retardation and organic diseases involving the central nervous system (e.g., trauma, tumors, cerebrovascular diseases, central nervous system infections, epilepsy, and neurodegenerative disorders); (3) were right-handed. We excluded patients who: (1) had severe mental illness/psychotic symptoms; (2) were undergoing electroconvulsive therapy, transcranial magnetic stimulation, and psychotherapy; (3) were pregnant or had MRI contraindications. Seventy-five HCs matched for age and gender were also recruited through community advertisements during the same period. HCs with a family history of psychiatric illnesses, history of mental retardation, and organic diseases involving the central nervous system were excluded.

The research protocol was approved by the First Affiliated Hospital of Kunming Medical University's ethics committee. All participants were provided with information about the study and informed consent was obtained.

### 2.2. Subgroups

Patients were divided into two groups according to the criteria from a previous study [24]. Specifically, patients were assigned to the MD group if they: (1) had a score ≥4 on the Montgomery–Asberg Depression Rating Scale (MADRS) item 8 (anhedonia); (2) scored ≥5 on the MADRS item 1 or 2 (nonreactive mood); (3) met at least three of the following criteria: HDRS item 2 score (guilt) ≥1, HDRS item 6 (late insomnia) scores ≥1, HDRS item 8 or 9 (psychomotor disturbance) score ≥1, and HDRS item 12 (appetite) or HDRS item 16 (weight loss) score = 2. Patients who did not meet the criteria above were assigned to the NMD group.

### 2.3. MRI Data Acquisition

MRI images were acquired from all participants using a Philips Achieva 3.0T MRI scanner, operated by a professional imaging technologist, at the imaging department of the First Affiliated Hospital of Kunming Medical University. Sponge pads were applied to the participants' heads during scanning to reduce movement. To exclude apparent structural abnormalities, conventional T1 and T2 MRIs were obtained. For patients with no apparent structural brain abnormalities on cranial planimetry, a 3D structural scan was performed with a fast perturbed phase gradient echo sequence. Repetition time = 7.38 ms, echo time = 3.4 ms, layer thickness = 1.2 mm, the field of view = 250 mm × 250 mm, matrix = 256 × 256, no interval, flip angle = 8°, number of layers = 230 layers, and scan time = 6 min 53 s.

### 2.4. VBM Analyses

DICOM images were converted into Neuroimaging Informatics Technology Initiative (NIfTI) format using the MRIconvert software (https://softpedia-secure-download.com/dl/8b4fdaeff4300d44a89816141cee5479/66f7807f/100181697/software/science/MRIConvert_235_Windows.zip). The CAT12 toolbox (http://dbm.neuro.Uni-jena.de/cat/) in the SPM12 software package (http://www.fil.ion.ucl.ac.uk/spm/software/spm12) was used for image preprocessing with the Matlab 2016a. The T1-weighted images were spatially normalized using the DARTEL algorithm [25], and segmented into three parts, gray matter (GM), white matter (WM), and cerebrospinal fluid (CSF). All segmented images were displayed in one slice. Sample homogeneity was checked to ensure the images are free of artifacts, misorientation, or preprocessing errors. Spatially normalized GM images for VBM were smoothed with full width at half maximum (FWHM) Gaussian kernel of 8 mm. Smoothed GM images were then used for VBM analysis.

### 2.5. Spatial Correlation Between Altered GMV of MD or NMD Patients With Receptor/Transporter Densities

We used the voxel-wise unthresholded *T* statistic maps of the *t*-test (depression-induced GMV changes) as the input for spatial correlation with the brain-wide spatial expression of receptors/transporters provided by the JuSpace toolbox [18, 19]. The PET- and SPECT-derived maps of the serotonin systems (5HT1A, 5HT1B, 5HT2a, 5HT4, and serotonin transporter (SERT)) [26, 27], the cannabinoid receptor (CB1r) [28], the dopamine systems (D1, D2, DAT, and FDOPA) [29–32], the GABAergic receptor [33], the *μ*-opioid receptor (MUr) [34], the noradrenaline transporter (NAT) [35], the vesicular acetylcholine transporter (VAChT) [36], and the metabotropic glutamate receptor 5 [37] were computed using Spearman correlations. Based on the Neuromorphometrics atlas, the spatial autocorrelation of local GM probabilities was adjusted, and the exact *p* value of spatial correlation was determined by permutation statistics with 10,000 permutations [18, 38]. Correlations were considered significant at *p*  < 0.05 [39, 40].

### 2.6. Statistical Analysis

One-way analysis of variance was used to analyze group differences in age and years of education among the three groups using SPSS26.0. We also performed a *χ*^2^ test to examine the gender. Meanwhile, a two-sample *t*-test was performed to analyze group differences in disease duration, HDRS_−17_, and MADRS scores between MD and NMD patients. The threshold *p* value less than 0.05 (two-sided) was considered a statistically significant difference.

An analysis of covariance (ANCOVA) was performed to compare the differences in GMV across the three groups using the CAT12 software, with age, gender, years of education, and TIV as covariates. Post hoc *t*-tests were performed to compare GMV differences between groups. Gaussian random field theory (GRF) was executed to correct multiple comparisons at *p*  < 0.05 (voxel significance: *p*  < 0.001 and cluster significance: *p*  < 0.05) [41].

Brain regions with abnormal GMV in MD patients compared to NMD patients were identified as the regions of interest (ROI). Then, we extracted the values of the ROI regions in MD patients for correlation analyses. Pearson correlations and Bonferroni corrections were used to examine the correlation between disease severity and the extracted GMV values in MD patients.

## 3. Results

### 3.1. Demographic and Clinical Characteristics

Three MD patients and one NMD patient were excluded due to quality issues with their MRI T1 data. At last, 63 patients with MD and 70 with NMD were included in the study. No statistical differences were found in age and gender among the three groups. We also did not see significant differences in the illness durations between the MD and NMD groups. Meanwhile, patients with MD (*p*=0.048) and NMD (*p*=0.001) showed significantly shorter education duration than HCs. However, there are no significant differences in education durations between patients with MD and NMD (*p*=0.223). Additionally, the total scores of HDRS_−17_ (*p*  < 0.001) and MADRS (*p*  < 0.001) were significantly higher in the MD group compared to the NMD group (Table 1).

### 3.2. Differences in GMV Between Groups

**Figure 1:**
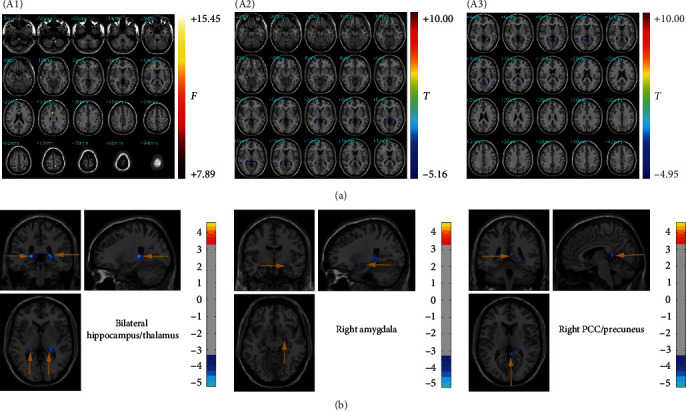
(A) (A1) Statistical maps showing ANOVA result of GMV abnormalities among patients with MD or NMD and HCs. (A2) Brain region showing the GMV deficits in the MD group compared with HCs. (A3) Brain region showing the GMV deficits in the MD group compared with NMD. The color bar represents the *F*- or *T*-value. (B) Brain region showing the GMV deficits in the MD group compared with HCs. The color bar represents the *T*-value.

Results of the ANCOVA analysis of the GMV among the three groups are displayed in Table 2 and Figure 1A. The left precuneus, bilateral cerebelum_9, right hippocampus, right anterior cingulate cortex, right middle cingulate cortex, right middle temporal gyrus, and right angular gyrus were significantly different among the three groups. Compared to HCs, MD patients had a significant GMV reduction in the bilateral hippocampus, bilateral thalamus, right amygdala, and right PCC/precuneus. Compared to NMD patients, MD patients showed a significant GMV reduction in the bilateral PCC/precuneus and lateral occipital cortex. There were no statistically significant differences in GMV between NMD patients and HCs after correction. To enhance clarity, we have provided Figure 1B that shows and label the brain regions with GMV deficits in the MD group compared to the HC group in different anatomical planes.

### 3.3. The Correlation Between Abnormal GMV and Clinical Variables

The GMV of the left lateral occipital cortex, right lateral occipital cortex, and bilateral PCC/precuneus in the MD group were not correlated with the severity of the disease (HDRS_−17_: *r* = 0.08, *p*=0.531; *r* = −0.147, *p*=0.252; *r* = 0.055, *p*=0.666 and MADRS: *r* = 0.057, *p*=0.567; *r* = −0.154, *p*=0.227; *r* = −0.032, *p*=0.801, separately).

### 3.4. Relationship to Neurotransmitter Receptor/Transporter Density

**Figure 2:**
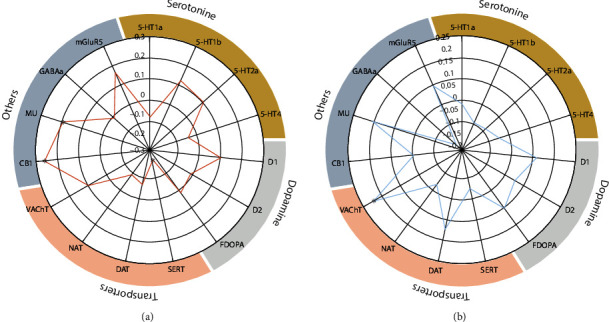
(A). Compared to HCs, the association between changes of GMV in MD patients with receptors/transporters densities. (B) Compared to NMD patients, the association between changes of GMV in MD patients with receptors/transporters densities (for *p* values, Table 3). The radial values are *r* values representing the spatial correlations. The “*⁣*^*∗*^” means the correlation was significant (*p*  < 0.05 for permutation, uncorrected). Note: 5-HT1a, 5-HT subtype 1a receptor; 5-HT1b, 5-HT subtype 1b receptor; 5-HT2a, 5-HT subtype 2a receptor; 5-HT4, 5-HT subtype 4 receptor; CB1, cannabinoid receptor; D1, dopamine D1 receptor; D2, dopamine D2 receptor; FDOPA, dopamine synthesis capacity; DAT, dopamine transporter; GABAa, GABAergic receptor subtype a receptor; mGluR5, metabotropic glutamate receptor 5; MU, μ-opioid receptor; NAT, noradrenaline transporter; SERT, serotonin transporter; VAChT, vesicular acetylcholine transporter.

Compared to HCs, changes of GMV introduced by MD were spatially correlated with the SERT, CB1r, and MUr. Compared to NMD patients, changes in GMV introduced by MD were spatially correlated with VAChT (Figure 2).

## 4. Discussion

### 4.1. Abnormal GMV Changes in Depression Patients With and Without Melancholic Features

The roles of the amygdala, hippocampus, and thalamus in the pathophysiological mechanisms of depression have been widely discussed in previous literature [42, 43]. Consistent with previous studies [9, 19, 44, 45], we also found GMV reduction in the hippocampus and thalamus among MD patients. However, our findings are inconsistent with previous studies [10, 19, 44, 46] regarding GMV alterations in the amygdala in patients with depression, which may be the result of the lack of classifications of depression subtypes in the previous studies. Other factors, including disease duration, age, and drug exposure, may also explain the inconsistency. Moreover, anhedonia was one of the core symptoms of MD and was associated with the dysfunction of the reward circuit [47, 48]. As components of the reward circuit, the hippocampus and amygdala could provide emotional, motivational information, and thalamic integrated information from the reward circuit [49]. In mood disorders, amygdala activity associated with increased negative emotions and the inability of the hippocampus to encode rewarding memories correctly may make individuals insensitive to positive information [50]. Moreover, disruptions of the thalamic response suggested that patients with MDD may have difficulties with action execution in response to rewarding cues [51]. Thus, the present study indicates that early GMV alterations in the brain regions among MD patients are associated with the reward circuit.

One interesting finding of the present study is that the GMV in PCC/precuneus decreased significantly in MD patients compared to NMD patients. The PCC/precuneus is an important node of the posterior default mode network (DMN) [52] and is related to problem-solving ability and memory function [10]. Several studies have found significant abnormalities in the DMN of MDD patients compared to HCs [53, 54]. Meanwhile, changes within the DMN and DMN connectivity patterns were associated with different ages, antidepressants, and specific depressive symptoms (e.g., hopelessness, rumination, or suicidality) [55], suggesting that changes in the structure or connectivity within the DMN may be closely associated with the specific clinical features of MDD. Meanwhile, a previous study found consistent results in the network homogeneity of the PCC/precuneus in MD and NMD patients [56]. Thus, the present study further confirms the significant differences in the structure of within DMN between MD and NMD patients, indicating that the structural abnormalities within the DMN might be associated with different subtypes of depression.

Additionally, we found a GMV reduction in the bilateral lateral occipital cortex in MD patients compared to NMD patients. The lateral occipital cortex is associated with visual processing processes [57]. In addition, previous studies found that visual processing regions were involved in the pathogenesis of MDD [58]. Furthermore, compared to NMD patients, MD patients had worse visual learning of cognitive function [59]. Therefore, we believe that the structural changes in the lateral occipital cortex are related to the worse visual cognitive processing in MD patients, but the mechanism needs to be further studied.

### 4.2. Association Between GMV Alterations and Molecular Architecture in Depression Patients With Melancholic Features

In the present study, we found that compared to HCs, changes in GMV introduced by MD were spatially associated with SERT, CB1r, and MUr. Previous literature has found sufficient evidence suggesting the critical role of monoaminergic neurotransmitter system dysfunction in the neurobiology of MDD. The reduced presynaptic release or abnormal signal transduction of monoamine neurotransmitters may lead to changed receptor regulation or function or impaired intracellular signaling, which might be the physiological changes behind these neurotransmitter signaling abnormalities [11]. Meanwhile, the SERT located on the axon terminal and soma of the serotonergic neurons is responsible for the reuptake of free 5-HT in the synaptic cleft [60, 61], which is also the mechanism of the action of selective serotonin reuptake inhibitors and serotonin–norepinephrine reuptake inhibitors. Selective serotonin reuptake inhibitors increase the concentration of extracellular 5-HT by blocking SERT's recapture of serotonin molecules, and thus acting as antidepressants or anxiolytics [61, 62]. Moreover, 5-HT also contributes to synapse formation, connectivity, and network construction during neuronal development. Specifically, 5-HT affects the neuronal plasticity of developing and developed brains by regulating cell adhesion molecules and BDNF [63–65]. A previous animal experiment demonstrated that knocking out the SERT gene in the nonserotonin-producing neurons in mice could lead to lasting changes in the spatial organizations of cortical neurons and dendritic arborization in the sensory cortex [66]. Several molecular imaging studies have found that MDD patients had decreased availability of SERT in the midbrain compared to HCs [67–69], which might be the result of the altered distribution of SERT and its abnormal function. Additionally, alterations in the interconnections between SERT and 5-HT can lead to abnormal neurodevelopment. Thus, it is unsurprising that SERT is associated with GMV alterations alterations. Moreover, previous studies found that MD and atypical depression were associated with the SERT gene promoter region [70, 71], suggesting an association between SERT and depression subtypes.

The endocannabinoid system can regulate the key neurological functions in the central nervous system, including mood regulation, motivated behavior, and cognitive function [72]. Meanwhile, CB1r, which mediates the physiological effects of endogenous cannabinoids in the central nervous system, is widely distributed in brain regions such as the cerebellum, hippocampus, and central amygdala [72, 73]. Previous studies showed that CB1r density in the anterior cingulate gyrus and frontal cortex changed in postmortem MDD patients [74, 75]. Moreover, endogenous opioid peptides and related receptors widely distributed in the brain's limbic and cortical regions regulated behavioral functions, including mood, emotion, motivation, and reward processing [76]. A retrospective study discovered that the subclinical symptoms of depression and anxiety were associated with lower MUr availability in the thalamus, amygdala, hippocampus, ventral striatum, and insular cortices [77]. Additionally, a PET study revealed that the relief of depressive symptoms after a week of placebo treatment was associated with increased placebo-induced μ-opioid neurotransmission in the network of regions in the subgenual anterior cingulate cortex, nucleus accumbens, midline thalamus, and amygdala [78]. In conclusion, previous studies highlighted the potential role of CB1r and MUr dysregulation in the pathophysiology and treatment of depression. However, to date, no evidence supports the association of CB1r and MUr with subtypes of depression. Therefore, further research is still needed.

Interestingly, in the present study, compared to NMD patients, changes in GMV introduced by MD were spatially associated with VAChT. Based on previous research, the activity and expression of VAChT significantly impacted the release of acetylcholine (Ach), which was associated with the symptoms of depression and anxiety [79]. A previous study indicated that stress might cause a chronic increase in the Ach levels in the hippocampus, which may cause synaptic plasticity in different neuronal subtypes and ultimately lead to mood disorders [80]. In addition, many previous studies found changes in VAChT in patients with diseases mainly characterized by altered cognitive function, including Alzheimer's disease and Lewy body dementia [81]. Since MD patients have more severe cognitive impairment than NMD patients [5], we hypothesize that VAChT may be closely associated with the melancholic features of MDD based on the findings of previous research and the current study.

The present study has several limitations. First, the case definitions of MD and NMD vary across different studies. In the current study, we defined them based on previous studies. Moreover, the present study is cross-sectional based on a single dataset. Therefore, studies with more datasets are still needed to further validate our results on the morphological changes in brain regions and the role played by neurotransmitters in the progression of the disease. Additionally, because the current study did not classify other subtypes of NMD, our results cannot comprehensively explain the pathogenesis of NMD. Finally, the data provided by the JuSpace toolbox were obtained from public datasets. In the present study, the spatial correlation analysis did not account for the difference of samples. Future studies should focus on assessing neurotransmitter receptor changes at the individual level.

In conclusion, the current study found that changes in GMV in brain regions are associated with distinct clinical features of MD patients. Moreover, we found that the neuroimaging changes associated with MD patients may be modulated by a specific neurochemical basis. The current study provided new insight into the pathological mechanisms, clinical features, and future therapeutic approaches to MD.

## Figures and Tables

**Table 1:** Demographic and clinical characteristics of all participants.

Variable	MD (*n* = 63)	NMD (*n* = 70)	HC (*n* = 75)	t/*χ*^2^	*p* value
Age (years)	36.05 ± 11.46	34.63 ± 9.64	36.03 ± 10.51	0.682	0.507^a^
Gender (male/female)	13/50	24/46	21/54	3.073	0.215^b^
Handedness (right/left)	63/0	70/0	75/0	—	—
Years of education (years)	11.79 ± 3.97	10.86 ± 4.74	13.29 ± 4.45	5.638	0.004^a^
Illness duration (months)	11.81 ± 18.82	11.82 ± 14.52	—	−0.003	0.997^c^
HDRS_−17_ scores	26.03 ± 4,50	21.37 ± 3.31	0.51 ± 0.71	6.847	<0.001^c^
MADRS scores	33.73 ± 6.37	25.74 ± 5.36	0.47 ± 0.96	7.844	<0.001^c^

Abbreviations: HC, healthy control; HDRS_−17_, 17-item Hamilton Depression Rating Scale; MADRS, Montgomery–Asberg Depression Rating Scale; MD, melancholic depression; NMD, nonmelancholic depression.

^a^The *p* value was obtained by analyses of variance.

^b^The *p* value was obtained by a *χ*^2^ test.

^c^The *p* value was obtained by a two-sample *t*-test.

**Table 2:** Regions of GMV deficits in MD or NMD compared with HC.

Cluster	Brain region	Voxels	MNI coordinates	*F* or *T* value
ANCOVA				*F* value
1	Left precuneus	105	−13, −43, 70	15.4508
2	Left cerebelum_9	105	−10, −47, −51	11.4027
3	Right hippocampus	33	27, −35, 5	9.8205
4	Right anterior cingulate cortex	122	15, 39, 9	10.4393
5	Right middle cingulate cortex	95	3, 10, 31	9.5888
6	Right middle temporal gyrus	23	67, −18, −11	8.3069
7	Right angular gyrus	39	56, −57, 31	9.2217
8	Right cerebelum_9	89	11, −47, −53	12.2218
MD vs. HC				*T* value
1	Left hippocampus	133	−22, −34, 6	−5.1666
	Left thalamus	—	—	—
2	Right hippocampus	315	22, −34, 6	−4.901
	Right thalamus	—	—	—
3	Right amygdala	31	27, −9, −15	−3.5244
4	Right PCC/precuneus	91	6, −43, 10	−4.8569
NMD vs. HC/MD vs. NMD				*T* value
1	Left lateral occipital cortex	56	−24, −75, 36	−3.903
2	Right lateral occipital cortex	69	33, −73, 24	−4.6489
3	Bilateral PCC/precuneus	303	−3, −47, 15	−4.9572

*Note*: GRF corrected: *p* < 0.05 (voxel significance: *p* < 0.001 and cluster significance: *p* < 0.05).

Abbreviations: ANCOVA, analyses of covariance; GMV, gray matter volume; MNI, Montreal Neurological Institute; PCC, posterior cingulate cortex.

**Table 3:** The associations between GMV components and neurotransmitter receptors/transporters activity maps.

Component PET map	Spearman's rho	*p* values (parametric)	*p* values (corrected)
GMV (MD vs. HC)			
5HT1a	−0.1243	0.180	0.4500
5HT1b	0.0988	0.286	0.5175
5HT2a	0.0812	0.380	0.5175
5HT4	−0.0895	0.334	0.5175
SERT	**−0.2466**	**0.008*⁣*^*∗*^**	**0.0600**
CB1	**0.2550**	**0.006*⁣*^*∗*^**	**0.0600**
D1	0.0816	0.378	0.5175
D2	−0.0525	0.570	0.6257
DAT	−0.1137	0.220	0.4714
FDOPA	−0.0289	0.755	0.7550
GABAa	−0.0506	0.584	0.6257
MU	**0.1831**	**0.048*⁣*^*∗*^**	**0.2400**
NAT	−0.1379	0.137	0.4110
VAChT	0.0757	0.414	0.5175
mGluR5	0.1492	0.108	0.4050
GMV (MD vs. NMD)			
5HT1a	−0.01	0.914	0.9140
5HT1b	−0.0784	0.397	0.7150
5HT2a	−0.0733	0.429	0.7150
5HT4	−0.0409	0.659	0.8019
SERT	−0.0459	0.620	0.8019
CB1	−0.0126	0.892	0.9140
D1	0.0973	0.294	0.7150
D2	0.0430	0.642	0.8019
DAT	0.1210	0.192	0.7150
FDOPA	0.0848	0.360	0.7150
GABAa	−0.1736	0.062	0.3100
MU	0.1762	0.058	0.3100
NAT	−0.0363	0.695	0.8019
VAChT	**0.2047**	**0.028*⁣*^*∗*^**	**0.3100**
mGluR5	0.0835	0.367	0.7150

*Note*: Bold values indicate that the spatial distribution of SERT, CB1, MU and VAChT correlates significantly with deficit brain regions compared to other neurotransmitter receptors.

*⁣* ^*∗*^Correlations were considered significant at *p*  < 0.05; *p* values (corrected) means FDR corrected.

## Data Availability

The data that support the findings of this study are available from the corresponding author upon reasonable request.

## References

[1] Dold M., Bartova L., Fugger G. (2021). Melancholic Features in Major Depression—A European Multicenter Study. *Progress In Neuro-Psychopharmacology & Biological Psychiatry*.

[2] Rudaz D. A., Vandeleur C. L., Gebreab S. Z. (2017). Partially Distinct Combinations of Psychological, Metabolic and Inflammatory Risk Factors Are Prospectively Associated with the Onset of the Subtypes of Major Depressive Disorder in Midlife. *Journal of Affective Disorders*.

[3] Yuan L., Chu Z., Chen X., Zhu Y., Xu X., Shen Z. (2023). Changes of Cortical Thickness in the First Episode, Drug-Naive Depression Patients With and Without Melancholic Features. *Psychiatry Research: Neuroimaging*.

[4] Tondo L., Vázquez G. H., Baldessarini R. J. (2020). Melancholic Versus Nonmelancholic Major Depression Compared. *Journal of Affective Disorders*.

[5] Valerio M. P., Lomastro J., Igoa A., Martino D. J. (2023). Neurocognitive Function of Patients With Melancholic and Non-Melancholic Major Depressive Episodes: An Exploratory Study. *Australian & New Zealand Journal of Psychiatry*.

[6] Guo C. C., Hyett M. P., Nguyen V. T., Parker G. B., Breakspear M. J. (2016). Distinct Neurobiological Signatures of Brain Connectivity in Depression Subtypes During Natural Viewing of Emotionally Salient Films. *Psychological Medicine*.

[7] Harald B., Gordon P. (2012). Meta-Review of Depressive Subtyping Models. *Journal of Affective Disorders*.

[8] Serra-Blasco M., Radua J., Soriano-Mas C. (2021). Structural Brain Correlates in Major Depression, Anxiety Disorders and Post-Traumatic Stress Disorder: A Voxel-Based Morphometry Meta-Analysis. *Neuroscience & Biobehavioral Reviews*.

[9] Hickie I., Naismith S., Ward P. B. (2005). Reduced Hippocampal Volumes and Memory Loss in Patients With Early- and Late-Onset Depression. *British Journal of Psychiatry*.

[10] Vassilopoulou K., Papathanasiou M., Michopoulos I. (2013). A Magnetic Resonance Imaging Study of Hippocampal, Amygdala and Subgenual Prefrontal Cortex Volumes in Major Depression Subtypes: Melancholic Versus Psychotic Depression. *Journal of Affective Disorders*.

[11] Liu Y., Zhao J., Guo W. (2018). Emotional Roles of Mono-Aminergic Neurotransmitters in Major Depressive Disorder and Anxiety Disorders. *Frontiers In Psychology*.

[12] Kraus C., Hahn A., Savli M. (2012). Serotonin-1A Receptor Binding Is Positively Associated With Gray Matter Volume—A Multimodal Neuroimaging Study Combining PET and Structural MRI. *NeuroImage*.

[13] Kraus C., Castrén E., Kasper S., Lanzenberger R. (2017). Serotonin and Neuroplasticity—Links Between Molecular, Functional and Structural Pathophysiology in Depression. *Neuroscience & Biobehavioral Reviews*.

[14] Kupfer D. J., Frank E., Phillips M. L. (2012). Major Depressive Disorder: New Clinical, Neurobiological, and Treatment Perspectives. *The Lancet*.

[15] Liu B., Liu J., Wang M., Zhang Y., Li L. (2017). From Serotonin to Neuroplasticity: Evolvement of Theories for Major Depressive Disorder. *Frontiers In Cellular Neuroscience*.

[16] Krystal J. H., Sanacora G., Blumberg H. (2002). Glutamate and GABA Systems as Targets for Novel Antidepressant and Mood-Stabilizing Treatments. *Molecular Psychiatry*.

[17] Kennis M., Gerritsen L., van Dalen M., Williams A., Cuijpers P., Bockting C. (2020). Prospective Biomarkers of Major Depressive Disorder: A Systematic Review and Meta-Analysis. *Molecular Psychiatry*.

[18] Dukart J., Holiga S., Rullmann M. (2021). JuSpace: A Tool for Spatial Correlation Analyses of Magnetic Resonance Imaging Data With Nuclear Imaging Derived Neurotransmitter Maps. *Human Brain Mapping*.

[19] Chen Y., Chen Y., Zheng R. (2023). Convergent Molecular and Structural Neuroimaging Signatures of First-Episode Depression. *Journal of Affective Disorders*.

[20] Chen J., Wei Y., Xue K. (2023). The Interaction Between First-Episode Drug-Naïve Schizophrenia and Age Based on Gray Matter Volume and Its Molecular Analysis: A Multimodal Magnetic Resonance Imaging Study. *Psychopharmacology*.

[21] Sakreida K., Chiu W.-H., Dukart J. (2022). Disentangling Dyskinesia from Parkinsonism in Motor Structures of Patients with Schizophrenia. *Brain Communications*.

[22] Montgomery S. A., Åsberg M. (1979). A New Depression Scale Designed to Be Sensitive to Change. *British Journal of Psychiatry*.

[23] Ma S., Yang J., Yang B. (2021). The Patient Health Questionnaire-9 Vs The Hamilton Rating Scale for Depression in Assessing Major Depressive Disorder. *Frontiers In Psychiatry*.

[24] Peters E. M., Zhang Y., Lodhi R., Li H., Balbuena L. (2021). Melancholic Features in Bipolar Depression and Response to Lamotrigine: A Pooled Analysis of Five Randomized Placebo-Controlled Trials. *Journal of Clinical Psychopharmacology*.

[25] Ashburner J. (2007). A Fast Diffeomorphic Image Registration Algorithm. *NeuroImage*.

[26] Savli M., Bauer A., Mitterhauser M. (2012). Normative Database of the Serotonergic System in Healthy Subjects Using Multi-Tracer PET. *NeuroImage*.

[27] Beliveau V., Ganz M., Feng L. (2017). A High-Resolution In Vivo Atlas of the Human Brain’s Serotonin System. *The Journal of Neuroscience*.

[28] Hansen J. Y., Shafiei G., Markello R. D. (2022). Mapping Neurotransmitter Systems to the Structural and Functional Organization of the Human Neocortex. *Nature Neuroscience*.

[29] Kaller S., Rullmann M., Patt M. (2017). Test-Retest Measurements of Dopamine D1-Type Receptors Using Simultaneous PET/MRI Imaging. *European Journal of Nuclear Medicine and Molecular Imaging*.

[30] Jaworska N., Cox S. M. L., Tippler M. (2020). Extra-Striatal D2/3 Receptor Availability in Youth at Risk for Addiction. *Neuropsychopharmacology*.

[31] Dukart J., Holiga Š., Chatham C. (2018). Cerebral Blood Flow Predicts Differential Neurotransmitter Activity. *Scientific Reports*.

[32] García-Gómez F. J., García-Solís D., Luis-Simón F. J. (2013). [Elaboration of the SPM Template for the Standardization of SPECT Images with 123I-Ioflupane]. *Revista Espanola De Medicina Nuclear E Imagen Molecular*.

[33] Nørgaard M., Beliveau V., Ganz M. (2021). A High-Resolution In Vivo Atlas of the Human Brain’s Benzodiazepine Binding Site of GABAA Receptors. *NeuroImage*.

[34] Turtonen O., Saarinen A., Nummenmaa L. (2021). Adult Attachment System Links With Brain Mu Opioid Receptor Availability In Vivo. *Biological Psychiatry*.

[35] Lobsien D., Müller U., Baldofski S. (2017). Central Noradrenaline Transporter Availability in Highly Obese, Non-Depressed Individuals. *European Journal of Nuclear Medicine and Molecular Imaging*.

[36] Aghourian M., Legault-Denis C., Soucy J. P. (2017). Quantification of Brain Cholinergic Denervation in Alzheimer’s Disease Using PET Imaging With [18F]-FEOBV. *Molecular Psychiatry*.

[37] Smart K., Cox S. M. L., Scala S. G. (2019). Sex Differences in [11C]ABP688 Binding: A Positron Emission Tomography Study of mGlu5 Receptors. *European Journal of Nuclear Medicine and Molecular Imaging*.

[38] Hirjak D., Schmitgen M. M., Werler F. (2022). Multimodal MRI Data Fusion Reveals Distinct Structural, Functional and Neurochemical Correlates of Heavy Cannabis Use. *Addiction Biology*.

[39] Cui S., Jiang P., Cheng Y., Cai H., Zhu J., Yu Y. (2023). Molecular Mechanisms Underlying Resting-State Brain Functional Correlates of Behavioral Inhibition. *NeuroImage*.

[40] Balajoo S. M., Eickhoff S. B., Masouleh S. K. (2023). Hippocampal Metabolic Subregions and Networks: Behavioral, Molecular, and Pathological Aging Profiles. *Alzheimer’s & Dementia*.

[41] Shan X., Cui X., Liu F. (2021). Shared and Distinct Homotopic Connectivity Changes in Melancholic and Non-Melancholic Depression. *Journal of Affective Disorders*.

[42] Diener C., Kuehner C., Brusniak W., Ubl B., Wessa M., Flor H. (2012). A Meta-Analysis of Neurofunctional Imaging Studies of Emotion and Cognition in Major Depression. *NeuroImage*.

[43] Zhang Y., Yang Y., Zhu L. (2020). Volumetric Deficit Within the Fronto-Limbic-Striatal Circuit in First-Episode Drug Naïve Patients With Major Depression Disorder. *Frontiers In Psychiatry*.

[44] Weniger G., Lange C., Irle E. (2006). Abnormal Size of the Amygdala Predicts Impaired Emotional Memory in Major Depressive Disorder. *Journal of Affective Disorders*.

[45] Grieve S. M., Korgaonkar M. S., Koslow S. H., Gordon E., Williams L. M. (2013). Widespread Reductions in Gray Matter Volume in Depression. *NeuroImage: Clinical*.

[46] Frodl T., Meisenzahl E. M., Zetzsche T. (2003). Larger Amygdala Volumes in First Depressive Episode as Compared to Recurrent Major Depression and Healthy Control Subjects. *Biological Psychiatry*.

[47] Leventhal A., Rehm L. (2005). The Empirical Status of Melancholia: Implications for Psychology. *Clinical Psychology Review*.

[48] Su Y.-A., Si T. (2022). Progress and Challenges in Research of the Mechanisms of Anhedonia in Major Depressive Disorder. *General Psychiatry*.

[49] Haber N. S., Knutson B. (2010). The Reward Circuit: Linking Primate Anatomy and Human Imaging. *Neuropsychopharmacology*.

[50] Russo S. J., Nestler E. J. (2013). The Brain Reward Circuitry in Mood Disorders. *Nature Reviews Neuroscience*.

[51] Yang X., Su Y., Yang F. (2022). Neurofunctional Mapping of Reward Anticipation and Outcome for Major Depressive Disorder: A Voxel-Based Meta-Analysis. *Psychological Medicine*.

[52] Utevsky A. V., Smith D. V., Huettel S. A. (2014). Precuneus Is a Functional Core of the Default-Mode Network. *The Journal of Neuroscience*.

[53] Greicius M. D., Flores B. H., Menon V. (2007). Resting-State Functional Connectivity in Major Depression: Abnormally Increased Contributions From Subgenual Cingulate Cortex and Thalamus. *Biological Psychiatry*.

[54] Scalabrini A., Vai B., Poletti S. (2020). All Roads Lead to the Default-Mode Network-Global Source of DMN Abnormalities in Major Depressive Disorder. *Neuropsychopharmacology*.

[55] Brakowski J., Spinelli S., Dörig N. (2017). Resting State Brain Network Function in Major Depression— Depression Symptomatology, Antidepressant Treatment Effects, Future Research. *Journal of Psychiatric Research*.

[56] Yan M., Cui X., Liu F. (2021). Abnormal Default-Mode Network Homogeneity in Melancholic and Nonmelancholic Major Depressive Disorder at Rest. *Neural Plasticity*.

[57] Mullin C. R., Steeves J. K. E. (2011). TMS to the Lateral Occipital Cortex Disrupts Object Processing but Facilitates Scene Processing. *Journal of Cognitive Neuroscience*.

[58] Zhao Y., Chen L., Zhang W. (2017). Gray Matter Abnormalities in Non-Comorbid Medication-Naive Patients With Major Depressive Disorder or Social Anxiety Disorder. *EBioMedicine*.

[59] Zaninotto L., Solmi M., Veronese N. (2016). A Meta-Analysis of Cognitive Performance in Melancholic Versus Non-Melancholic Unipolar Depression. *Journal of Affective Disorders*.

[60] Pourhamzeh M., Moravej F. G., Arabi M. (2022). The Roles of Serotonin in Neuropsychiatric Disorders. *Cellular and Molecular Neurobiology*.

[61] Sahu A., Gopalakrishnan L., Gaur N. (2018). The 5-Hydroxytryptamine Signaling Map: An Overview of Serotonin-Serotonin Receptor Mediated Signaling Network. *Journal of Cell Communication and Signaling*.

[62] Deo N., Redpath G. (2021). Serotonin Receptor and Transporter Endocytosis Is an Important Factor in the Cellular Basis of Depression and Anxiety. *Frontiers In Cellular Neuroscience*.

[63] Daubert E. A., Condron B. G. (2010). Serotonin: A Regulator of Neuronal Morphology and Circuitry. *Trends In Neurosciences*.

[64] Dalva M. B., McClelland A. C., Kayser M. S. (2007). Cell Adhesion Molecules: Signalling Functions at the Synapse. *Nature Reviews Neuroscience*.

[65] Duman R. S., Monteggia L. M. (2006). A Neurotrophic Model for Stress-Related Mood Disorders. *Biological Psychiatry*.

[66] Chen X., Ye R., Gargus J. J., Blakely R. D., Dobrenis K., Sze J. Y. (2015). Disruption of Transient Serotonin Accumulation by Non-Serotonin-Producing Neurons Impairs Cortical Map Development. *Cell Reports*.

[67] Joensuu M., Tolmunen T., Saarinen P. I. (2007). Reduced Midbrain Serotonin Transporter Availability in Drug-Naïve Patients With Depression Measured by SERT-Specific [(123)I] Nor-Beta-CIT SPECT Imaging. *Psychiatry Research*.

[68] Parsey R. V., Hastings R. S., Oquendo M. A. (2006). Lower Serotonin Transporter Binding Potential in the Human Brain During Major Depressive Episodes. *The American Journal of Psychiatry*.

[69] Hsieh P. C., Chen K. C., Yeh T. L. (2014). Lower Availability of Midbrain Serotonin Transporter Between Healthy Subjects With and Without a Family History of Major Depressive Disorder—A Preliminary Two-Ligand SPECT Study. *European Psychiatry*.

[70] Willeit M., Praschak-Rieder N., Neumeister A. (2003). A Polymorphism (5-HTTLPR) in the Serotonin Transporter Promoter Gene Is Associated With DSM-IV Depression Subtypes in Seasonal Affective Disorder. *Molecular Psychiatry*.

[71] Baune B. T., Hohoff C., Mortensen L. S., Deckert J., Domschke V. A. (2008). Serotonin Transporter Polymorphism (5-HTTLPR) Association With Melancholic Depression: A Female Specific Effect?. *Depression and Anxiety*.

[72] Navarrete F., García-Gutiérrez M. S., Jurado-Barba R., Rubio G., Gasparyan A. (2020). Endocannabinoid System Components as Potential Biomarkers in Psychiatry. *Frontiers In Psychiatry*.

[73] Matsuda L. A., Lolait S. J., Brownstein M. J., Young A. C., Bonner T. I. (1990). Structure of a Cannabinoid Receptor and Functional Expression of the Cloned cDNA. *Nature*.

[74] Koethe D., Llenos I. C., Dulay J. R. (2007). Expression of CB1 Cannabinoid Receptor in the Anterior Cingulate Cortex in Schizophrenia, Bipolar Disorder, and Major Depression. *Journal of Neural Transmission*.

[75] Choi K., Le T., McGuire J. (2012). Expression Pattern of the Cannabinoid Receptor Genes in the Frontal Cortex of Mood Disorder Patients and Mice Selectively Bred for High and Low Fear. *Journal of Psychiatric Research*.

[76] Jelen L. A., Stone J. M., Young A. H., Mehta M. A. (2022). The Opioid System in Depression. *Neuroscience & Biobehavioral Reviews*.

[77] Nummenmaa L., Karjalainen T., Isojärvi J. (2020). Lowered Endogenous Mu-Opioid Receptor Availability in Subclinical Depression and Anxiety. *Neuropsychopharmacology*.

[78] Peciña M., Bohnert A. S. B., Sikora M. (2015). Association Between Placebo-Activated Neural Systems and Antidepressant Responses: Neurochemistry of Placebo Effects in Major Depression. *JAMA Psychiatry*.

[79] Higley M. J., Picciotto M. R. (2014). Neuromodulation by Acetylcholine: Examples From Schizophrenia and Depression. *Current Opinion In Neurobiology*.

[80] Drever B. D., Riedel G., Platt B. (2011). The Cholinergic System and Hippocampal Plasticity. *Behavioural Brain Research*.

[81] Kanel P., Bedard M.-A., Aghourian M. (2021). Molecular Imaging of the Cholinergic System in Alzheimer and Lewy Body Dementias: Expanding Views. *Current Neurology and Neuroscience Reports*.

